# Resection and repair of postoperative diaphragmatic hernia with strangulated bowel obstruction after esophageal cancer via thoracic cavity: A case report

**DOI:** 10.1097/MD.0000000000043894

**Published:** 2025-08-15

**Authors:** Zhen Li, Ling Zong, Na Li, Ziteng Zhang, Ronghang Hu, Baobin Xu, Dongwei Wang, Lu Ning, Qichen Liang

**Affiliations:** a Department of Thoracic Surgery, Affiliated Hospital of Jining Medical University, Jining, Shandong, PR China; b Department of Thoracic Surgery, Qinghai Red Cross Hospital, Xining, Qinghai, PR China; c School of Clinical Medicine (Affiliated Hospital), Jining Medical University, Jining, Shandong, PR China.

**Keywords:** case report, esophageal cancer, postoperative diaphragmatic hernia, strangulated intestinal obstruction

## Abstract

**Rationale:**

Postoperative diaphragmatic hernia is an uncommon complication following esophagectomy for esophageal cancer. Once it occurs, emergency surgical intervention is typically required. However, the optimal surgical repair approach remains debatable depending on the specific clinical scenario. Therefore, we aim to investigate the common etiological factors and optimal surgical strategies for post-esophagectomy diaphragmatic hernia, and share our therapeutic experience to provide optimal treatment for patients.

**Patient concerns:**

A 70-year-old male patient developed sudden chest tightness and retrosternal pain during nocturnal sleep 17 months after radical esophagectomy, accompanied by persistent pain in the left upper abdomen that prevented standing, with nausea (no vomiting), and absence of defecation or flatus.

**Diagnoses:**

Diaphragmatic hernia, strangulated intestinal obstruction, and esophageal cancer (postoperative status).

**Interventions:**

Emergency transthoracic diaphragmatic hernia repair with partial small bowel resection was performed via thoracic approach.

**Outcomes:**

On postoperative day 9, the patient’s bowel and urinary functions returned to normal, resumed a semiliquid diet, was discharged after suture removal.

**Lessons:**

Post-esophagectomy diaphragmatic hernia represents a severe complication. Open transthoracic surgery proves to be a safe and effective approach for cases complicated by strangulated intestinal obstruction. However, in cases of diaphragmatic hernia following minimally invasive esophagectomy without preoperative evidence of strangulation, laparoscopic exploration and repair could be considered. Regardless of open or minimally invasive esophagectomy, meticulous operative techniques should be employed to minimize the risk of diaphragmatic hernia formation.

## 1. Introduction

Esophagus cancer is a common malignant tumor of the digestive system, and according to global cancer statistics in 2022, there were 511,000 cases of esophagus cancer and 445,000 deaths, ranking 11th and 7th, respectively, among all malignant tumors. The annual incidence of esophageal cancer in China is 224,000 cases, with a crude incidence rate of 15.87 per 100,000 and an age-standardized incidence rate of 8.32 per 100,000, ranking 7th among all malignant tumors; and the annual death rate is 187,500 cases, with a crude death rate of 13.28 per 100,000 and an age-standardized death rate of 6.68 per 100,000, ranking 5th among all malignant tumors.^[1]^ Esophageal cancer is characterized by its high degree of invasiveness and unfavorable prognosis, with a 5-year survival rate of <30% in the majority of countries, thus constituting a significant threat to global health. The optimal approach to managing this condition is radical resection. However, it should be noted that during surgery, the normal physiology of the body is disrupted, and one of the uncommon but fatal complications is postoperative diaphragmatic hernia. This refers to the herniation of abdominal organs into the thoracic cavity through the diaphragmatic incision or through the esophageal hiatus due to a sharp rise in intra-abdominal pressure. Postoperative diaphragmatic hernia has been reported in the aftermath of open esophagectomy, total minimally invasive esophagectomy and hybrid minimally invasive esophagectomy, with incidence rates ranging from 0% to 28%.^[2–5]^ Postoperative diaphragmatic hernias manifest either early in the postoperative period or months or even years later. When they do occur, emergency surgery is often required. However, the optimal surgical intervention for addressing these hernias remains a subject of debate, contingent on the prevailing circumstances at the time of the hernia’s development.^[6]^ Therefore, we present a case report of a patient who underwent emergency thoracic approach for primary resection of necrotic intestine and repair of diaphragmatic hernia following left thoracotomy for radical esophagectomy, resulting in successful recovery. This case is reported to investigate the common etiologies of postoperative diaphragmatic hernia and explore appropriate surgical approaches, with the aim of optimizing therapeutic strategies for affected patients.

## 2. Case report

### 2.1. Medical history

A 70-year-old male patient was emergently admitted to the Department of Thoracic Surgery at Jining Medical University Affiliated Hospital on November 24, 2024, due to “sudden onset of chest pain and chest tightness lasting for 1 day.” The patient had undergone a “left thoracotomy for radical esophagectomy” at a local hospital in June 2023, with uneventful postoperative recovery. One day prior to admission, the patient experienced abrupt-onset chest pain and chest tightness accompanied by persistent left upper abdominal pain during nocturnal sleep. The pain rendered him unable to stand and was associated with nausea (without vomiting), as well as absence of defecation and flatus.

### 2.2. Physical examination

Vital signs: T: 36.0°C, P: 117 beats/min, R: 20 breaths/min, BP: 113/67 mm Hg. The patient was alert and oriented with normal mental status but exhibited an acute distress facial expression. No palpable supraclavicular lymphadenopathy was noted bilaterally. The thorax was normoconfigured without deformities. Diminished breath sounds were auscultated in the left lung field, while the right lung field demonstrated coarse breath sounds. Cardiac examination revealed no abnormal precordial bulging or thrills. The heart rhythm was regular, and no pathological murmurs were detected. The abdomen was flat and soft on palpation, with tenderness localized to the left upper quadrant but no rebound tenderness.

### 2.3. Auxiliary examinations

**Figure 1.**
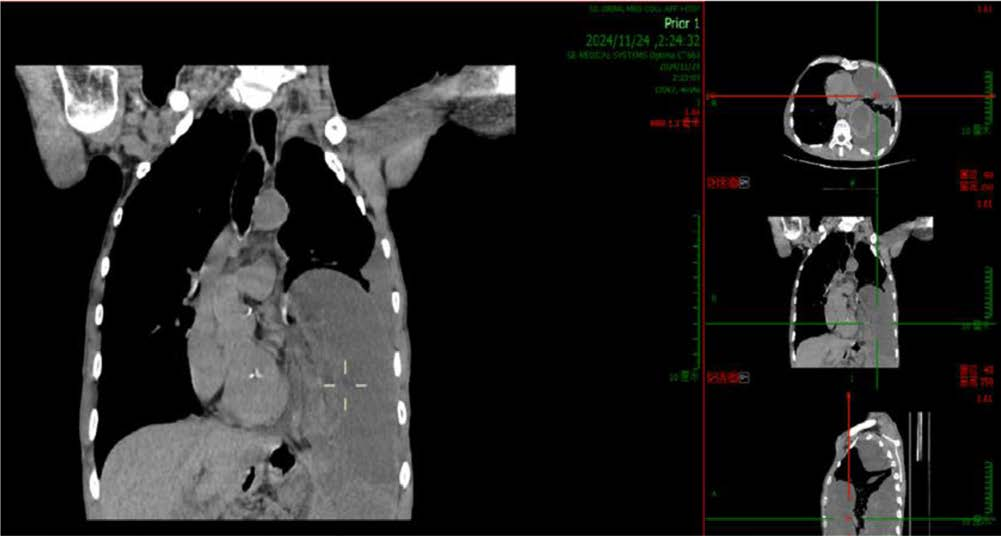
November 24, 2024, chest and abdominal CT demonstrated a left-sided diaphragmatic hernia complicated by bowel obstruction. CT = computed tomography.

November 24, 2024, complete blood count + C-reactive protein: White blood cell count: 12.99 × 10^9^/L, C-reactive protein: 3.5 mg/L. Urgent liver function test: Albumin: 40.4 g/L. Thoracic and abdominal computed tomography: (1) Postoperative changes of esophageal carcinoma; (2) Left diaphragmatic hernia complicated by partial intestinal obstruction and left pulmonary atelectasis; (3) Left pleural effusion; and (4) Retention of colonic contents (Fig. 1).

### 2.4. Medical course of diagnosis and treatment

Based on the patient’s medical history, physical examination, and ancillary test results, the diagnosis was postoperative diaphragmatic hernia with strangulated intestinal obstruction following left transthoracic esophageal cancer surgery. The patient underwent emergency surgery on November 24, 2024, at 5:18 am under combined intravenous-inhalation anesthesia, which included left transthoracic diaphragmatic hernia repair and partial small bowel resection.

#### 2.4.1. Intraoperative details

**Figure 2.**
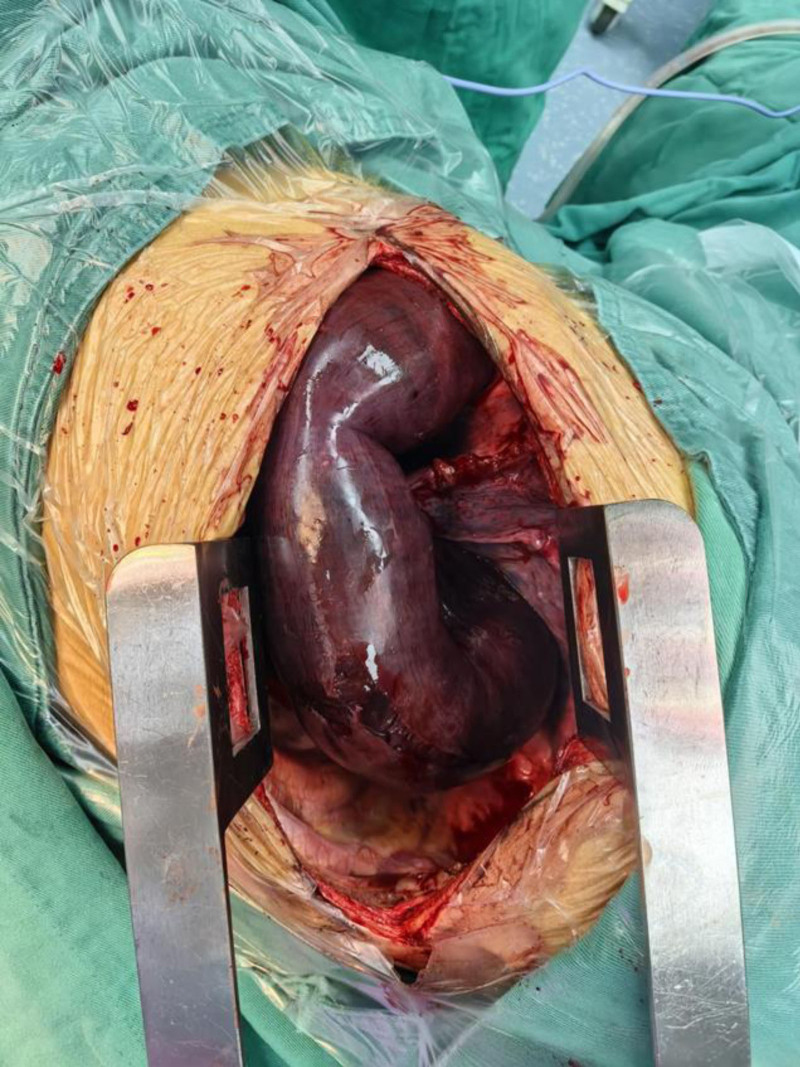
Herniated bowel loops within the thoracic cavity were severely dilated, tension-filled, and displayed patchy discoloration.

The patient was placed in the right lateral decubitus position. A left posterolateral thoracotomy incision was made, sequentially dissecting through the skin, subcutaneous tissue, latissimus dorsi, serratus anterior, trapezius, and intercostal muscles, followed by entry into the pleural cavity via the 7th intercostal space. The thoracic cavity exhibited localized adhesions and bloody effusion. Herniated bowel loops within the thoracic cavity were severely dilated, tension-filled, and displayed patchy discoloration (Fig. 2).

**Figure 3.**
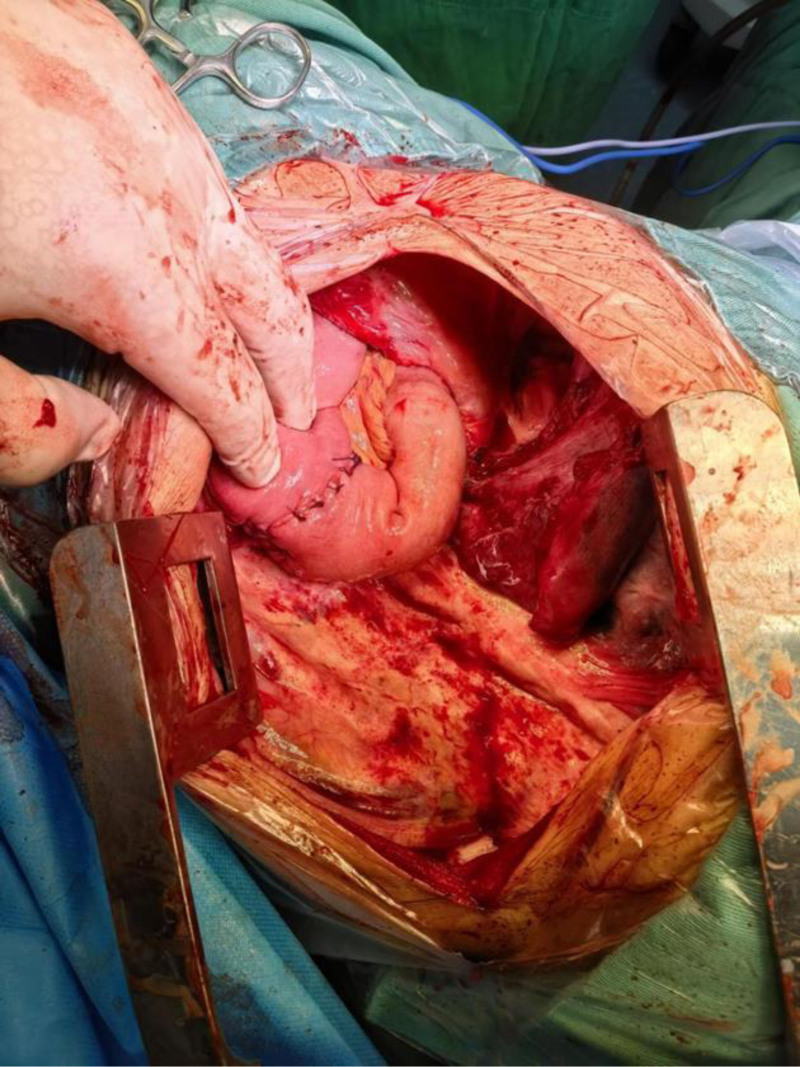
A side-to-side anastomosis was performed between the proximal and distal ends of the small bowel.

**Figure 4.**
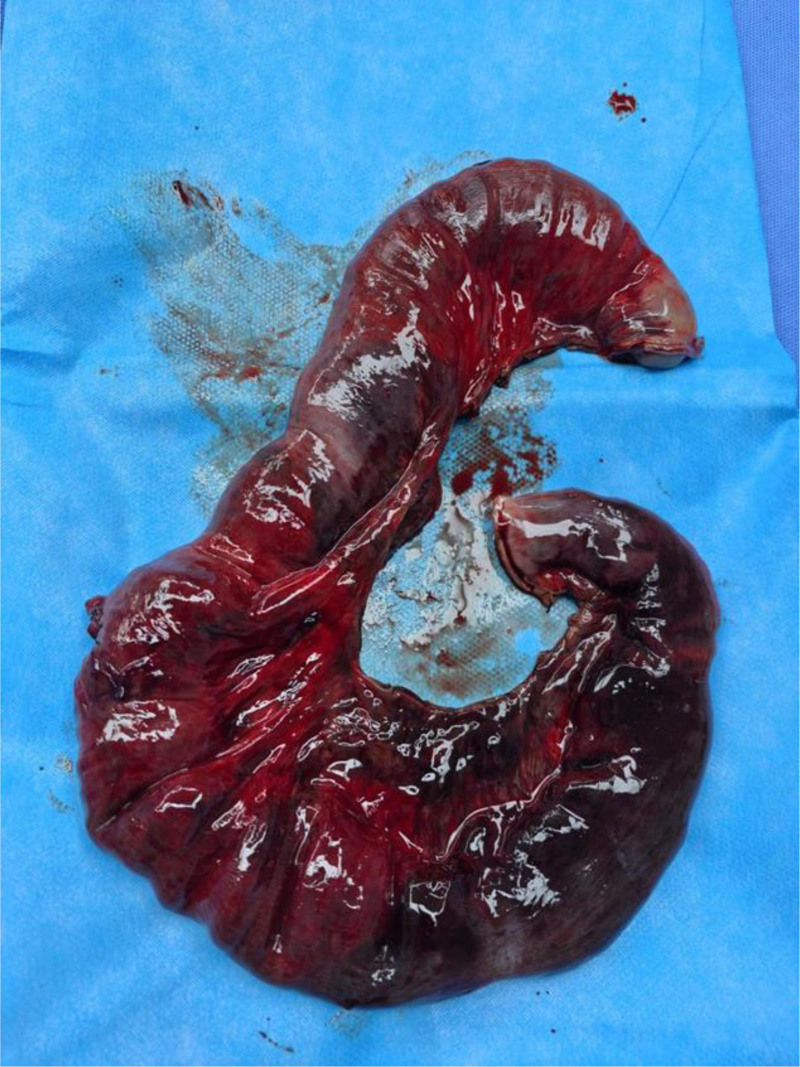
A 30-cm segment of ischemic small bowel (jejunum) was identified, showing characteristic signs of transmural necrosis including loss of peristalsis, greenish-black discoloration, and absence of pulsations in mesenteric vessels. The necrotic segment was resected using linear staplers.

Intraoperative findings confirmed diaphragmatic hernia and intestinal necrosis. Due to advanced necrosis, extreme tension, and thinned bowel walls, manipulation led to bowel perforation, resulting in massive spillage of intestinal contents into the thoracic cavity. The cavity was thoroughly irrigated with copious warm saline, and the perforation was closed using a purse-string suture. Negative pressure suction was applied to evacuate residual effusion and decompress the incarcerated bowel. Adhesiolysis of the lung was performed, revealing a 2-cm diaphragmatic defect located 2 cm from the reconstructed diaphragmatic hiatus, through which the bowel had herniated. The diaphragmatic defect was extended by incising the surrounding tissue, exposing ischemic necrosis of the corresponding mesentery. A definitive diagnosis of strangulated intestinal obstruction and small bowel necrosis was established. Via the diaphragmatic incision, the abdominal cavity was explored. The necrotic small bowel segment was identified approximately 30 cm distal to the ligament of Treitz, spanning 30 cm in length. The mesentery was dissected, intermittently ligated, and divided using an energy device until reaching viable tissue. The necrotic bowel was transected with a linear stapler, and the stump was oversewn. A side-to-side anastomosis was performed between the proximal and distal ends of the small bowel (Figs. 3 and 4).

**Figure 5.**
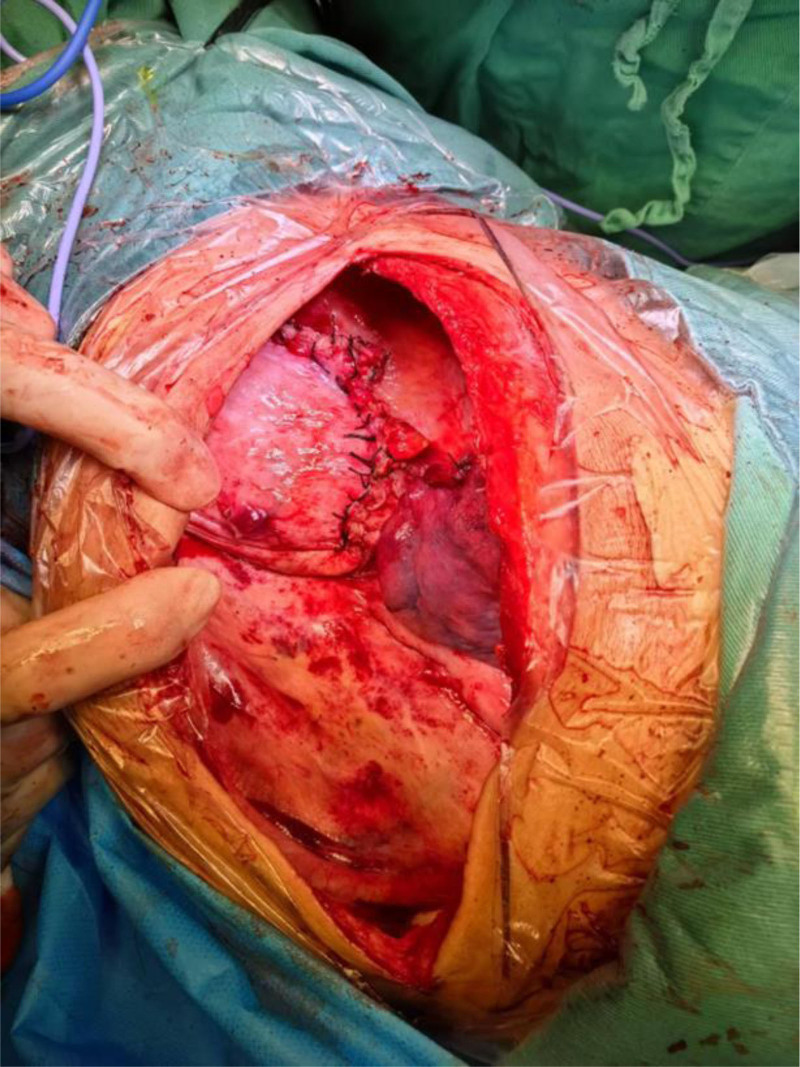
The anastomosis was oversewn with interrupted sutures, the mesenteric defect was closed, the diaphragmatic incision was repaired, and the diaphragmatic hiatus was reconstructed.

The anastomosis was reinforced with interrupted sutures, and the mesenteric defect was closed. The diaphragmatic incision was repaired, and the diaphragmatic hiatus was reconstructed (Fig. 5).

#### 2.4.2. Operative report

The thoracic cavity was repeatedly irrigated with povidone–iodine saline solution. After achieving complete hemostasis, a chest tube was placed in the 9th intercostal space at the midaxillary line, and the incision was closed in layers. The procedure was completed successfully with an estimated blood loss of 50 mL. Intraoperative fluid administration totaled 3300 mL, with urine output of 500 mL. No blood transfusion was required. The patient was transferred to the ward postoperatively and maintained on NPO status with intravenous nutritional support and antibiotic therapy. Oral intake was planned to resume gradually after recovery of bowel function.

#### 2.4.3. Progress notes

November 25, 2024, 8:00 am (postoperative day [POD] 1): The patient reported reduced chest/abdominal pain but persistent incisional pain (score 2/10). Complaints included mild cough with scant white sputum. No dyspnea, chest tightness, or flatus. Vital signs stable. Exam: Alert, intact chest dressing without exudate, coarse breath sounds bilaterally with scattered wet rales. Abdomen soft, non-tender. Bowel sounds absent.

November 26, 2024, 8:00 am (POD 2): Incisional pain decreased (1/10). Flatus achieved. Chest tube drained 430 mL pale red exudate. Coarse breath sounds with reduced rales. Labs: WBC 10.17 × 10^9^/L, RBC 2.71 × 10^12^/L.

November 27, 2024, 8:00 am (POD 3): Incisional pain (1/10). Productive cough with white sputum. Chest tube output decreased to 310 mL serosanguinous fluid. NPO continued with IV nutrition and antibiotics.

**Figure 6.**
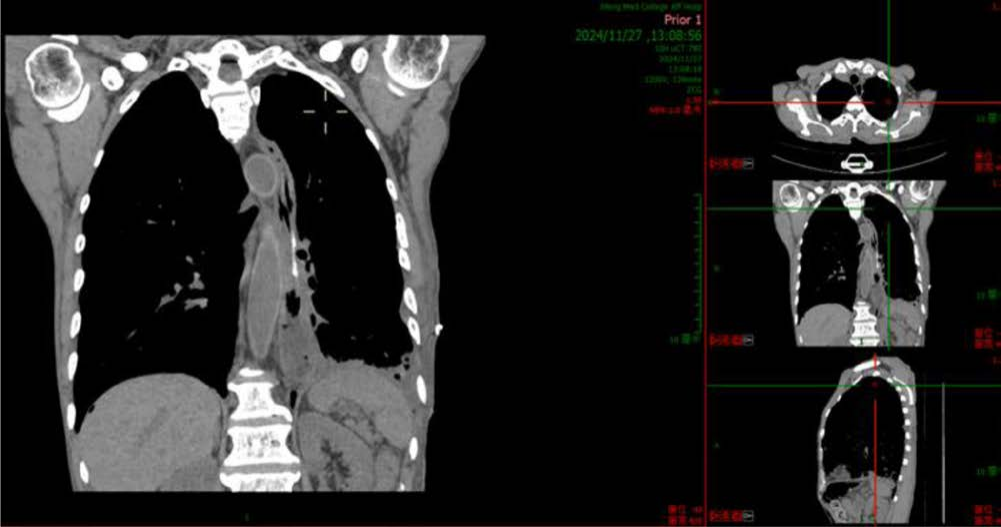
November 28, 2024, follow-up CT demonstrated postoperative changes from diaphragmatic hernia repair and partial small bowel resection, along with prior esophagectomy, with no evidence of diaphragmatic hernia recurrence. CT = computed tomography.

November 28, 2024, 8:00 am (POD 4): Hemoptysis noted (blood-tinged sputum), likely related to intraoperative pulmonary injury. Minimal stool after enema. Chest drainage reduced to 150 mL amber fluid. Tolerated clear liquids, advanced to full liquid diet. Computed tomography findings: (1) Postoperative changes from diaphragmatic hernia repair + partial enterectomy. (2) Status post-esophagectomy. (3) Bilateral pulmonary inflammation (Fig. 6).

December 3, 2024, 8:00 am (POD 9): Pain-free (0/10). Occasional blood-tinged sputum persists. Transitioned to semiliquid diet. Incision healing well without erythema/exudate. Plan: Suture removal tomorrow. Discharge instructions included high-protein diet, small frequent meals, head elevation during sleep, and oral antibiotics. Follow-up scheduled in 1 month.

The patient provided written informed consent for the publication of this case report.

## 3. Discussion

With the significant improvement in survival rates among patients undergoing esophagectomy for esophageal cancer, postoperative diaphragmatic hernia has emerged as an increasingly prominent complication,^[7,8]^ even as the surgical approach has evolved from open procedures to MIE. The introduction of MIE has substantially enhanced surgical outcomes, reducing intraoperative blood loss, pulmonary complications, postoperative pain, and hospital stay duration.^[9]^ However, the incidence of diaphragmatic hernia following both surgical approaches remains nearly equivalent.^[10]^ A meta-analysis of 6058 patients revealed a higher incidence of diaphragmatic hernia after MIE (4.5%) compared to open esophagectomy (1.0%), though the etiologies differ between the 2 techniques. In open esophagectomy, hernias typically arise at the diaphragmatic repair site due to excessively wide suture spacing or insecure knotting. In contrast, MIE-associated hernias predominantly occur at the esophageal hiatus, often resulting from intraoperative widening of the hiatus during dissection of the distal esophagus and cardia without prophylactic repair.^[2,11,12]^ The standardization of surgical management for postoperative diaphragmatic hernia remains controversial. Some authors advocate laparoscopic repair as the primary approach, while others propose alternative strategies.^[13,14]^ The choice of surgical approach depends on the phase of pathology: for chronic lesions, the transthoracic approach is preferred due to its superior diaphragmatic exposure and feasibility for adhesiolysis, which aligns with the demands of meticulous reconstruction.^[15]^ Although current studies show no significant association between surgical approach and complications (e.g., pneumonia, surgical site infection) or mortality, thoracoscopic diaphragmatic hernia repair remains linked to higher recurrence rates and longer operative times compared to open approaches.^[16]^ Herein, we report a case of a patient with postoperative diaphragmatic hernia and strangulated intestinal obstruction following esophageal cancer resection, successfully managed via transthoracic necrotic bowel resection and hernia repair, followed by uneventful recovery. This case highlights the advantages of transthoracic exploration and repair. Compared to other approaches, this method directly addresses the diaphragmatic defect, restoring diaphragmatic integrity to prevent visceral re-herniation and reduce complications. Additionally, it allows simultaneous management of intestinal obstruction through the hiatus, such as resection of necrotic bowel or adhesiolysis. While the transabdominal approach enables visualization of herniated contents and adjacent gastric conduit vasculature, minimizing iatrogenic injury,^[3,17]^ it fails to assess intrathoracic herniated structures. In this case, the herniated small intestine and mesentery were necrotic and under high tension, posing a significant risk of rupture. Transabdominal exploration would have risked intrapleural contamination from potential rupture, leading to severe consequences. Transthoracic exploration, however, may face challenges in patients with prior left thoracotomy due to dense pleural adhesions, making minimally invasive thoracic approaches impractical. Open thoracotomy via the 7th intercostal space (avoiding the original 6th intercostal incision) effectively circumvents adhesions. The transthoracic approach minimizes intra-abdominal organ manipulation, reducing adhesion-related complications. Postoperative adhesions often render abdominal approaches insufficient for diaphragmatic hernia repair. Based on this case, open transthoracic repair is a safe and effective strategy for strangulated intestinal obstruction. For MIE-associated hernias without strangulation, laparoscopic repair via the esophageal hiatus is feasible, as these hernias typically involve the enlarged hiatus and are amenable to reduction and hiatal closure under laparoscopy.

In this case, the patient’s history of esophageal cancer surgery likely caused disruption of diaphragmatic anatomy and depletion of nutritional reserves. Postoperative chronic malnutrition (e.g., hypoalbuminemia) and subclinical inflammation may further compromise tissue regenerative capacity, increasing the risk of diaphragmatic hernia formation and subsequent incarceration/strangulation of the small bowel.^[18,19]^ Therefore, integrating indicators such as prognostic nutritional index, neutrophil-to-lymphocyte ratio, and platelet-to-lymphocyte ratio into preoperative risk assessment protocols can help identify high-risk patients and optimize perioperative management (e.g., targeted nutritional support or anti-inflammatory interventions).^[20,21]^ This approach may reduce the incidence of such rare yet critical complications.

## 4. Conclusion

Postoperative diaphragmatic hernia is a severe complication of esophagectomy, necessitating prompt diagnosis and intervention to prevent life-threatening outcomes. Prophylaxis remains paramount. Surgeons should meticulously reconstruct the diaphragmatic hiatus during open esophagectomy, ensuring tight suture spacing and secure knots. In MIE, intraoperative evaluation of the esophageal hiatus is critical; excessive widening or laxity due to dissection of the esophagus or cardia should prompt immediate repair. These measures significantly reduce the risk of postoperative diaphragmatic hernia.

## Author contributions

**Investigation:** Ling Zong.

**Resources:** Zhen Li, Baobin Xu.

**Supervision:** Zhen Li, Ronghang Hu, Lu Ning.

**Visualization:** Na Li.

**Writing – original draft:** Zhen Li, Ziteng Zhang, Qichen Liang.

**Writing – review & editing:** Ling Zong, Na Li, Ronghang Hu, Baobin Xu, Dongwei Wang.
